# Honoring the Work of Robert McMahon: a Commentary on This Special Issue

**DOI:** 10.1007/s11121-025-01807-w

**Published:** 2025-04-21

**Authors:** Mark T. Greenberg

**Affiliations:** 1 Pennsylvania State University, University Park, PA 16802 USA; 2 Stanwood, WA 98292 USA

**Keywords:** Family interventions, Prevention, Longitudinal research

## Abstract

This commentary reviews papers prepared for this Special Issue on the work of Robert McMahon, who served as Editor of *Prevention Science* and who has played a major role on multiple aspects of parenting research and its relation to conduct problems in children and youth. The Special Issue papers are discussed in the context of the four main foci of Dr. McMahon’s work: (1) parenting practices and child development; (2) risk and protective factors in the development and maintenance of conduct problems; (3) family-based treatments for conduct problems; and (4) multicomponent preventive interventions for conduct problems. The papers emphasize the interplay between family dynamics, parenting practices, and developmental trajectories and underscore the importance of considering these factors within the parent the child and the social and cultural context. A clear finding across multiple papers is the importance of inhibitory control and emotion regulation in both predicting conduct problem outcomes across time for children as well as an important treatment focus for improving parenting. These papers re-emphasize McMahon’s research, which underscores the importance of early, sustained, and contextually sensitive parenting interventions for promoting lifelong positive outcomes in children and families, as well as the need for longitudinal studies that can reflect these pathways across time. This richly packed issue of papers in honor of Bob McMahon’s research and long-term Editorship of *Prevention Science* is quite remarkable, and it is an honor to write this commentary as his friend and colleague of over 35 years. The papers here focus on four key areas of Dr. McMahon’s research career, which focused on multiple aspects of parenting and the development of conduct problems and psychopathology. Broadly speaking, McMahon’s work can be divided into more basic and more applied, treatment-focused research. His more basic work centers on the understanding of how conduct problems (and other problem behaviors) develop in children and adolescents, their developmental course over time, and how various risk and protective factors influence their manifestation and stability. On the intervention side, from early in his career his persistent interest was how to effectively prevent and treat these problems with a focus on parenting interventions. As the primary family focused researcher in the (Conduct Problems Prevention Research Group, The Fast Track program for children at risk: Preventing antisocial behavior, Guilford Press, 2020), he led the Fast Track intervention work on the development and efficacy of a parenting intervention within the context of a large, multicomponent intervention for children with early, serious conduct problems that is still being studied more than 30 years later. His outstanding, multifaceted career involved both basic and applied research on parenting but always with a focus on how research can impact and improve practice. Here, I review these papers in the light of the four main foci of McMahon’s work: (1) parenting practices and child development; (2) risk and protective factors in the development and maintenance of conduct problems; (3) family-based treatments for conduct problems; and (4) multicomponent preventive interventions for conduct problems.

## Parenting Practices and Child Development

McMahon’s research emphasizes the role of parenting behaviors (e.g., coercive discipline and parental monitoring) in shaping child conduct problems and other developmental outcomes. Dr. McMahon formed a long-term partnership with his mentor Rex Forehand beginning in 1975 and published the first edition of *Helping the NonCompliant Child* in 1981. A major contribution of McMahon and Forehand’s early work on parenting was to focus not only parental discipline strategies but also on parental warmth and how the joint effects of improving warmth and discipline led to improving family atmosphere and family cohesion.

In this issue, Zheng and colleagues (2024) explored the daily relationships between parenting behaviors and adolescent conduct problems and callous-unemotional (CU) traits. Using daily diary data from 86 adolescents, the study highlighted several key findings: positive parenting behaviors (warmth and non-harsh discipline) were linked to reductions in CU traits on subsequent days. This finding provides some direction for universal parenting programs showing that CU traits are at least somewhat modifiable by context. However, as the study did not examine harsh discipline and relied on a middle-class sample with low rates of adolescent problem behavior, these variables require study in more diverse and clinical samples.

In their intergenerational family study, Fosco and colleagues (2024) examined the interplay between family climate and parental warmth and discipline across adolescence and linked these influences to parenting by these same youth in early adulthood in the longitudinal PROSPER study of rural adolescents. The study found reciprocal effects between positive family climate and effective discipline across adolescence, with family climate predicting early adult antisocial behavior and early adult antisocial behavior predicting next generation parental warmth. In addition, the results showed 1 st generation (G1) family climate predicted 2nd generation (G2) family conflict. This finding and a previous report that G1 positive family climate in adolescence predicted lower relationship violence in early adulthood (Xia et al., 2018) demonstrate that adolescent family climate has long-term effects on later family relationships and has clear implications for cross-generational prevention.

Following a similar theme, Lansford and colleagues (2024) examined key aspects of parenting across eight different world cultures. Echoing the previous two studies, they found that positive parenting was predictive of low rates of early conduct problems across all eight cultures. This finding of the effect of positive parenting supports models of early and continued secure attachment that focus on positive, responsive caregiving as a key for human development.

## Risk and Protective Factors in the Development and Maintenance of Conduct Problems

McMahon’s work has illustrated how ethnicity, neighborhood, and other contextual factors may influence the early and continued development of conduct problems. However, this body of work also recognized how the child’s own contributions may impact parenting and children’s pathways to healthy or unhealthy outcomes. In a landmark paper from the multisite Family Check-Up® (FCU) project for preschoolers, Suarez and colleagues (2024) examined the impact of inhibitory control at age 10 on conduct problems in adolescence. Importantly, they showed that caregiver ratings of inhibitory control are the sole unique predictors of later antisocial behavior. Further, they reported interaction effects such that inhibitory control had even stronger impacts on later conduct problems in the context of dangerous neighborhood environments and engaging with deviant peers. Inhibitory control, a key dimension of executive function, has long shown such effects. For example, Riggs et al. (2006) found that inhibitory control was the key for reducing aggression using the universal school-based PATHS curriculum (Greenberg et al., 2011).

A key question in the conduct problems field is the role of high vs. low CU traits, with children with both high conduct problems and CU traits showing greater risk for lifelong difficulties. The paper by Kemp et al. (2024) examines CU traits and emotion recognition deficits. In a normative sample of children, they reported an age by high CU trait difference, with adolescents indicating those high in CU traits showed better recognition of sad faces as compared to elementary aged children who had poorer recognition of sad faces. While the potential explanation is that with age children high in CU traits are more motivated to understand others’ emotions, this runs contrary to the idea that emotion difficulties are definitional in CU traits. Future studies require assessment of other aspects of emotion including emotion regulation. Further, to more fully differentiate conduct problems from CU traits would require larger sample sizes at each age (given their correlation of 0.48), in order to have a sufficient number of children who are high in conduct problems but not CU traits and vice versa.

Although positive parenting is clearly a key factor is reducing child and adolescent conduct problems, McMahon’s extensive work also has focused on defining and studying the effects of parental monitoring in different populations (Dishion & McMahon, 1998; Pelham et al., 2024; Racz & McMahon, 2011). Bellamy and colleagues (2024) have extended this work by examining three domains of psychopathic personality, of which CU traits is one of the domains. In a cross-sectional study, they report that adolescents with high CU traits disclose less information to parents, complicating effective parental monitoring. Small effects linked youth self-reported arousal to parental monitoring, and the authors imply that tailored interventions targeting psychophysiological factors (e.g., arousal) could reduce risks for conduct problems. Surely, interventions that focus on parent monitoring should be a priority for youth prevention of conduct problems, but it is not clear how interventions should be tailored to the level of child and adolescent self-reported arousal.

## Family-based Treatments for Conduct Problems

McMahon’s early work with Forehand in developing and testing the “Helping the Noncompliant Child” (HNC) treatment model has become a trusted intervention for supporting parenting and reducing aggressive behavior (Forehand & McMahon, 1981; McMahon & Forehand, 2003). Here, Highlander et al. (2024) validated its effectiveness in a study with low SES families. Using the Family System Model, they showed that within low-SES families, high financial strain diminished the effects of HNC at 12-month outcomes. While financial strain may be the key factor, causing direct effects, it may be that financial strain is a proxy for or interacts with other factors such as lack of social support, attachment issues, depressive affect, and insularity via the work of Wahler (1980) and others; further work is indicated to explore such exogenous variables.

Three papers in this issue focus on the impacts of the FCU intervention, which is a brief intervention/assessment parent model that incorporates a strength-based approach to human development (Dishion et al., 2012) at different ages. In the Suarez et al. (2024) paper discussed above, the results showed that using the FCU model with low-income families of preschoolers did not directly impact antisocial behavior a decade later but did show indirect effects via its effect on inhibitory control in childhood. In a second paper on FCU, Stormshak et al. (2024) report on two different trials that used a digital health version of FCU with parents of 10–14 year-olds with behavioral concerns. Using integrative data analysis across studies, they did not show a direct effect on conduct problems 6 months later but did show indirect effects via emotional problems and effortful control. Using a similar parent measure to Suarez et al., they replicated the impact of effortful or inhibitory control in altering risky behaviors. This study of a digital health model is an important addition to the field and the authors note the importance of individual online coaching to the success of the model. Finally, Bennett and colleagues (2024) provide a careful replication of FCU in a Canadian context with parents of 2–4 year-olds, closely replicating the significant findings to the original US study (Dishion et al., 2008). This preschool period is important for early intervention, before conduct problems become entrenched in the child and the parent–child relationship. Interestingly, 86% of FCU families participated in additional sessions that were offered, but there was no report of service usage in the comparison group. The finding that parental stress was not reduced suggests that offering sessions on parents’ own emotion regulation and coping with the challenges of parenting would be beneficial.

Regarding online programs, Canário et al. (2024) reported a meta-analysis of online parenting programs that raises as many questions as it answers, as it uses a small group of very diverse studies in terms of child age, baseline conduct problems, whether professional guidance is offered, and the model (universal, selective, and indicated prevention). As most of the studies included behavioral parent training as a primary component, it is not surprising that it was found to be an important component. But the question of what really works, for whom, at what ages, with what level of child problems, and with what socioeconomic and educational level of the parents — is still unknown (e.g., McMahon et al., 2021). Further, the conclusion that online programs are effective is premature given the above issues. As a recent paper from the same group concluded: “Study 1 shows that *at least when professional guidance is present*, online parenting support is an effective, non-inferior alternative to in-person support … An important shared feature of the online programs … is interaction between parents and professionals” (p. 7; Leijten et al., 2024).

The concern about using online parenting without therapist support for families with substantial needs is echoed in the wide-ranging and insightful paper by Turner and Sanders (2024) that reviews decades of work using the Triple P-Positive Parenting Program. Over several decades, Sanders and colleagues developed and evaluated a suite of parenting interventions using a tiered system of five levels from light touch to the needs of highly-impacted families in different cultural settings using face-to-face training, workbooks, online training, television shows, etc. It is a remarkable set of models, interventions, and studies. The results have been quite positive, and this has spread the Triple P system widely. Turner and Sanders made two most important observations. First, they emphasized the importance of a systems-contextual approach that embeds parenting not only in family systems, but in the larger systems of extended families, neighborhoods, and the exosystem of beliefs about parenting and the role of government services in the life of families. Second, echoing papers in this issue, they noted the key factor of parents’ regulatory and emotional control as central to treatment and change that supports children’s development. So, just as children’s inhibitory control skills are key for their pathways across life, parents’ own regulatory skills are key to treatment and the mechanisms of change.

## Multicomponent Preventive Interventions for Conduct Problems

As it is unlikely that a single intervention component alone (parenting, peer intervention, classroom management, and social-emotional learning programs) would be sufficient for altering the life-course pathway of children who are both poor and highly aggressive and defiant at school entrance, the idea of developing multicomponent interventions has had a long, but underdeveloped history in prevention research. With origins in the Cambridge study (McCord, 1978) from the 1930 s, it was not until the 1990 s that NIMH, under a special initiative, funded two multicomponent studies of children living in disadvantaged neighborhoods and having high aggressive and oppositional behavior at school entrance. Both of these studies are represented in this Special Issue.

The first, Fast Track, was led by a multi-university consortium in which both Dr. McMahon and I participated. Now in its 35 th year, the Fast Track project is the longest running of these multicomponent models. The results from when participants were age 25 years old were summarized in book form (Conduct Problems Prevention Research Group, 2020) and many articles have been published on both the developmental model and intervention outcomes (https://fasttrackproject.org/). In the current Special Issue, McCabe et al. (2024) examine direct and indirect effects of the Fast Track intervention at age 31. Although main effects were found at age 25, here they report on new outcomes at age 31 and show no significant direct positive effects of Fast Track. They do show numerous indirect effects on adult outcomes including fewer externalizing, internalizing, and substance use problems; reduced criminality and sexual partners; and increased general health and full-time employment. Thus, the Fast Track interventions that included a focus on friendships, conflict resolution, and getting along with others did have a long-term impact in adulthood. There is much more to investigate here including possible sex differences in adulthood, as Rothenberg et al. (2022) found that at age 34, Fast Track mothers had lower depression symptoms, alcohol problems, drug problems, corporal punishment use, and food insecurity compared to control group mothers, but there were no effects on fathers. Further, given differences in both urban/rural status and ethnicity, sub-analyses by site should be explored. Finally, subsequent analyses might employ latent profile models to examine participants at different levels of risk and at different developmental periods.

Another paper reports a second but relatively short-term multicomponent intervention (Prinz et al., 2024). This intervention also included a parent, peer, early reading, and classroom behavior interventions implemented in grades 1 and 2 for children with very high rates of early conduct problems. Prinz et al. (2024) reported that although there was no overall main effect, intervention boys showed higher teacher-reported reading grades in grade 3. In addition, there were no effects on either teacher or parent report of conduct problems by grade 3. It is unfortunate that funding was not available to continue the intervention and follow-up as larger effects might have been found, as seen in the Montreal Longitudinal Study that showed economic and social benefits in adulthood (Algan et al., 2022).

### Future Considerations for the Field

Here, I briefly discuss a few future considerations that arise from the papers of this issue and other recent findings. One essential consideration regards a broadening of the scope of measurement and intervention in parenting. As McMahon’s work has clearly illustrated, models of parenting need to focus on both warm and responsiveness as well as managing behavior via parent management techniques. As this issue illustrates, there is a need to consider differences in developmental trajectories and treatment responsiveness dependent on not only the severity of conduct problems but comorbidities including CU traits and internalizing problems, as well as parent and family well-being (e.g., depression and other psychopathological symptoms). As Frick (2024) has noted, children with high CU traits, including a surfeit of positive emotions, are likely to need a larger dose of support for facilitating parental warmth and responsiveness. Getting the balance right between parental responsiveness and parent management leads one to see a “one size fits all” solution to be ineffective. As any clinician knows, some “cases” will take more treatment sessions and have more potential for relapse than others. Thus, there is a need for more careful assessment and new randomized trials that provide more flexibility (given theory) in response to assessment findings — this is clearly one of the great advantages of the FCU model. As we know from many developmental studies of the attachment process, being able to assess/diagnose the issues around child vs. parent contributions to conduct problems as early as possible is likely to lead to better treatment effects (Narayan et al., 2021; Shelleby & Shaw, 2014). Further, multigenerational considerations of “ghosts in the nursery” may lead to a necessary focus on parental emotional schemas and cognitions as both a mediator of change and assessment of short-term effectiveness, as illustrated by early attachment interventions (Bernard et al., 2012) as well as those during adolescence (Moretti et al., 2024).

A second broad consideration is that of outcomes for children. While reducing the rate of conduct problems is of course a central goal of these efforts, there is a need to consider much broader aspects of the child’s well-being, including their emotional schema and cognitions, executive functions/self-regulatory skills, and peer relations. As one considers these broader issues of child well-being, one naturally considers the importance of interventions that not only include the family context but also school and peer contexts (Conduct Problems Prevention Research Group, 1992). While Fast Track and Prinz et al. (2024) are examples of such multilevel prevention/intervention models, there has been very limited multicontext intervention research for children with conduct problems and innovations that combine school and home interventions, especially in the early years, are needed.

A third broad consideration is where and how technology and distance learning may be advantageous and cost-effective. While it is quite unlikely (in my opinion) that most families with children with conduct problems will show substantial benefit only from an online program, online programs with strong initial assessment, flexibility in modules, and effective coaching by well-trained interventors may be quite effective in some cases. What will be most important here will be to understand for whom such hybrid programs may be effective. Given the high dropout rates in all forms on online learning, knowing for whom online/hybrid programs are not effective and having sufficient funds for other more traditional treatment models will be essential. It is highly likely that those family systems with the highest rate of chaos and family disorder/violence will need to build trust with highly skilled clinicians and see at least initial success before hybrid programs will be successful. Using online technologies effectively in this domain will require collaboration with clinics, schools, and families to find the right combinations for families with different needs (Aldridge et al., 2024).

In summary, the articles in this Special Issue honor Bob McMahon’s contributions and reflect the wide range of his influence on the research and practices of parenting. They emphasize the interplay between family dynamics, parenting practices, and developmental trajectories and underscore the importance of these factors within the parent, the child, and the social and cultural context. Further, as reflected in McMahon’s own work, this issue highlights the need for continued research that emphasizes the importance of early, sustained, and contextually sensitive interventions for promoting lifelong positive outcomes in children and families, and the need for longitudinal studies that can reflect these pathways across time (McMahon, 1994).
